# Do psychotic symptoms predict future psychotic disorders in adolescent psychiatry inpatients? A 17-year cohort study

**DOI:** 10.1017/S003329172500073X

**Published:** 2025-04-03

**Authors:** Valentina Kieseppä, Ulla Lång, Colm Healy, Kirstie O’Hare, Covadonga M. Díaz-Caneja, Sinan Gülöksüz, Bart P. F. Rutten, Mary Cannon, Anu-Helmi Halt, Pirkko Riipinen, Ian Kelleher

**Affiliations:** 1 Centre for Clinical Brain Sciences, Division of Psychiatry, University of Edinburgh, Edinburgh, UK; 2 Research Unit of Clinical Medicine, Faculty of Medicine, University of Oulu, Oulu, Finland; 3 Department of Public Health and Welfare, Finnish Institute for Health and Welfare, Helsinki, Finland; 4 School of Medicine, University College Dublin, Dublin, Ireland; 5 Discipline of Psychiatry and Mental Health, University of New South Wales, Sydney, Australia; 6 Department of Child and Adolescent Psychiatry, Institute of Psychiatry and Mental Health, Hospital General Universitario Gregorio Marañón, Instituto de Investigación Sanitaria Gregorio MaraÑón (IiSGM), CIBERSAM, ISCIII, School of Medicine, Universidad Complutense, Madrid, Spain; 7 Department of Psychiatry and Neuropsychology, Mental Health and Neuroscience Research Institute, Maastricht University, Faculty of Health, Medicine and Life Sciences, Maastricht, the Netherlands; 8 Department of Psychiatry, Yale School of Medicine, New Haven, CT, USA; 9 Department of Psychiatry, RCSI University of Medicine and Health Sciences, Dublin, Ireland; 10 Future Neuro Research Ireland Centre, RCSI University of Medicine and Health Sciences, Dublin, Ireland; 11 Department of Psychiatry, Oulu University Hospital, Oulu, Finland; 12 St. John of God Hospitaller Services Group, Hospitaller House, Dublin, Ireland

**Keywords:** child and adolescent mental health services, inpatient treatment, psychosis, psychotic symptoms, transition

## Abstract

**Background:**

Individuals with a psychiatric inpatient admission in adolescence have a high risk of schizophrenia-spectrum disorders (SSDs) when followed to adulthood. Whether psychotic symptoms predict subsequent SSDs in inpatient cohorts, however, is an important unanswered question.

**Methods:**

The sample consisted of adolescents (aged 13–17) admitted to psychiatric inpatient care (Oulu, Finland) from April 2001 to March 2006. Psychotic symptoms were assessed with the Schedule for Affective Disorders and Schizophrenia. Specialized health care use and diagnoses were followed up in national health care registers until June 2023. Cox regression was used to predict SSDs by the presence of baseline psychotic symptoms.

**Results:**

Of 404 adolescent inpatients admitted with non-psychotic mental disorders, 28% (*n* = 113) reported psychotic symptoms: 17% (*n* = 68) subthreshold and 11% (*n* = 45) full threshold. By the end of follow-up, 23% of the total cohort went on to be diagnosed with an SSD. Subthreshold psychotic symptoms did not differentiate patients who would subsequently develop SSDs (cumulative incidence 24%; HR = 1.42, 95%CI = 0.81–2.50). Full-threshold psychotic symptoms, on the other hand, were associated with an increased risk of subsequent SSDs (cumulative incidence 33%; HR = 2.00, 95%CI = 1.12–3.56). Most subsequent SSDs (83%), however, occurred in individuals who had not reported threshold psychotic symptoms during inpatient admission.

**Conclusions:**

There was a high risk of subsequent SSDs among adolescent psychiatry inpatients when followed over time. SSDs were not predicted by subthreshold psychotic symptoms. Full-threshold psychotic symptoms were associated with an increased risk of subsequent SSDs, though with low sensitivity.

## Introduction

Psychosis prediction has been a major focus of psychiatric research for decades (Fusar-Poli et al., 2020; Lindgren, Kuvaja, Jokela, & Therman, 2023; Oliver et al., 2022). The dominant approach to identifying psychosis risk has been to use symptomatic assessments of (predominantly subthreshold or attenuated) psychotic symptoms, such as the Comprehensive Assessment of at Risk Mental States (CAARMS) or the Structured Interview for Psychosis Risk Syndromes (SIPS) (Addington et al., 2019; Ajnakina et al., 2017; Ciarleglio et al., 2019; Gandhi & Cullen, 2022; Oliver et al., 2022; Polari et al., 2018).

Subthreshold psychotic symptoms are highly prevalent among adolescents hospitalized with non-psychotic mental disorders, with as many as one in four (non-psychotic) inpatients meeting criteria for DSM-5’s attenuated psychosis syndrome (APS) (Gerstenberg et al., 2015; Salazar de Pablo et al., 2020). Furthermore, it has previously been shown that adolescent psychiatry inpatients have a very high risk of subsequent psychotic disorders when followed to adulthood: in a cohort study of all individuals born in Finland in 1987 followed to age 28, 24% of all individuals with a psychiatric inpatient admission in childhood or adolescence went on to receive a diagnosis of a psychotic disorder by age 28 (Lång et al., 2022). Whether psychotic symptoms help to differentiate inpatients who will go on to develop psychotic disorders from those who will not, however, has not been tested to date. We carried out the first longitudinal study on the risk of psychotic disorder associated with subthreshold and threshold psychotic symptoms in an adolescent inpatient psychiatry sample.

Our study is part of a prospective clinical follow-up project (Ilomäki et al., 2008), which recruited adolescents treated in Oulu University Hospital’s adolescent psychiatry ward, a regional inpatient unit serving the two northernmost provinces of Finland (the provinces of Oulu and Lapland, covering 43% of the area of Finland) between years 2001 and 2006. Following the psychiatric inpatient admission in adolescence, all subsequent outpatient visits and inpatient stays with specialist mental health services, including associated psychiatric diagnoses, were captured up to June 2023 using the national Finnish Care Register for Health Care (FCRHC). Among study participants hospitalized for non-psychotic disorders, we calculated the cumulative risk of subsequent psychotic disorder when followed up for 17–22 years (up to ages 29 to 39 years old). We tested whether (1) subthreshold psychotic symptoms or (2) full threshold psychotic symptoms, predicted a higher risk of subsequent psychotic disorder compared to inpatients who had not reported psychotic symptoms.

## Method

### Study sample

All new adolescent patients who were admitted at a psychiatric inpatient ward at Oulu University Hospital, Department of Psychiatry (hereafter referred as index hospitalization), between April 2001 and March 2006 (*n* = 637) were invited to participate in the study (Ilomäki et al., 2008). Adolescents aged over 18 years (*n* = 1) and those diagnosed with an intellectual disability (*n* = 26), or an organic brain disorder (*n* = 3) were excluded. In addition, adolescents whose inpatient stay was too short for their interviews to be completed (*n* = 22) were also excluded from the study. For 77 individuals, either the guardians or the patients themselves declined to give consent to participate. Out of all eligible adolescents (*n* = 607), 508 (83.7%) participated.

All study participants and their legal guardians provided written informed consent for participation in the study. The project was approved by the Ethics Committee of the Oulu University Hospital and the Finnish Social and Health Data Permit Authority Findata has granted permission to use the personal register data (THL/4612/14.02.00/2022).

### Psychotic symptoms

Adolescents were assessed using the Schedule for Affective Disorder and Schizophrenia for School-Age Children – Present and Lifetime (K-SADS-PL) (Kaufman et al., 1997), a semi-structured interview assessing for a wide range of mental health disorders. Most of the interviews were conducted by trained medical student research assistants under the supervision of a physician. The remaining interviews were conducted directly by the treating physician.

For the current study, scores from the psychotic symptom screening section were used as the measure of subthreshold and threshold psychotic symptoms. This section consists of two items on hallucinations (probes include, e.g., ‘Has there ever been a time you heard voices when you were alone?’) and delusions (probes include, e.g., ‘Did you believe in things that other people didn’t believe in?’). The current presence of the symptoms was assessed by the interviewer who scored each item from 1 to 3 (1 = Symptom not present, 2 = Subthreshold level of symptomology, 3 = Threshold level of symptomology). Subthreshold symptoms were defined as symptoms that are not sufficient to count toward the diagnosis of a disorder, as judged by the interviewer, whereas threshold psychotic symptoms involve full threshold (i.e. without intact reality testing) hallucinations or delusions. The score was based on information derived from the participant. A combination variable of these two scores was formed to take value 3 if the highest score of either item was 3, to take value 2 if the highest score of either item was 2, and to otherwise take value 1.

### Registered diagnoses of psychotic disorders

Information on visits and hospital admissions in public specialized health care was retrieved from the FCRHC register. We retrieved information on the date of the visit/admission and the associated diagnoses at discharge. Information on inpatient treatments covered the whole lifetime of each participant, while data on outpatient visits in specialized levels of health care was available from the year 1998 onwards. Both inpatient and outpatient data are available until June 2023. Diagnostic codes are based on the 10th revision of the International Statistical Classification of Diseases and Related Health Problems (ICD-10) for visits from 1996 onwards, and on the 9th revision of the International Statistical Classification of Diseases and Related Health Problems (ICD-9) before 1996. The FCRHC register is considered highly reliable for research purposes (Sund, 2012).

Diagnosis of SSD was defined as any F20–F29 diagnosis based on ICD-10 and any of the following diagnostic codes based on ICD-9: (1) 295 Schizophrenia, (2) 297 Delusional disorder, and (3) 298 Other psychotic disorder. Both primary and secondary diagnoses were considered. All individuals who had a registered SSD diagnosis before or at their discharge date from the index hospitalization were excluded (*n* = 95).

Most individuals who received a validated psychotic disorder diagnosis based on K-SADS-PL diagnostic interview also had a registered diagnosis of psychosis in the FCRHC register before or at their discharge date from index hospitalization (*n* = 61), but nine individuals had a validated psychotic disorder diagnosis based only on the K-SADS-PL diagnostic interview. These individuals were also excluded, resulting in a final sample of 404 participants. A participant flow chart is available in Supplementary Figure S1.

### Other variables of interest

Information on *age at index hospitalization*, *sex, and duration of index hospitalization* (days) was retrieved from the initial study database. Information on *validated baseline diagnoses* was based on the K-SADS-PL interview performed during index hospitalization. The following six dichotomous categories were formed, based on the five diagnostic supplements provided by K-SADS-PL, each taking values ‘Yes’ and ’No’: the presence of (1) psychotic disorder, (2) anxiety disorder, (3) affective disorder, (4) substance use disorder, (5) conduct disorder, and (6) other psychiatric disorder. Information on a potential *date of death* was retrieved from the National Causes of Death register maintained by Statistics Finland.

### Statistical analysis

Descriptive statistics are reported for the total sample and when stratified by inclusion status in Table 1. Cumulative incidences of diagnoses of SSDs after discharge were calculated separately for those without psychotic symptoms, with subthreshold psychotic symptoms, and with threshold psychotic symptoms in the psychosis section of the K-SADS-PL.

**Table 1.** Descriptive statistics of the sample at baseline

	Total (*n* = 508)	Included: (*n* = 404) no previous or baseline psychosis	Excluded: (*n* = 104) previous or baseline psychosis
Sex (female) (*n* [%])	300 (59 %)	244 (60%)	56 (54%)
Age (mean [SD])	15.5 (1.3)	15.4 (1.3)	15.7 (1.3)
Duration of admission in days (mean [SD])	26.9 (63.6)	16.5 (30.9)	67.8 (118.8)
K-SADS-PL validated diagnoses at baseline (*n* [%])			
Psychotic disorder	70 (14%)	0 (0%)	70 (67%)
Anxiety disorder	117 (23%)	100 (25%)	17 (16%)
Affective disorder	251 (49%)	227 (56%)	24 (23%)
Substance use disorder	195 (38%)	169 (42%)	26 (25%)
Conduct disorder	225 (44%)	199 (49%)	26 (25%)
Other disorder	47 (9%)	38 (9%)	9 (9%)
Overall psychotic symptoms			
1 (Not present)	323 (64%)	288 (72%)	35 (34%)
2 (Subthreshold)	79 (16%)	68 (17%)	11 (11%)
3 (Threshold)	103 (20%)	45 (11%)	58 (56%)
Hallucinations			
1 (Not present)	358 (71%)	312 (78%)	46 (45%)
2 (Subthreshold)	66 (13%)	53 (13%)	13 (13%)
3 (Threshold)	81 (16%)	37 (9%)	44 (43%)
Delusions			
1 (Not present)	385 (77%)	341 (85%)	44 (43%)
2 (Subthreshold)	56 (11%)	44 (11%)	12 (12%)
3 (Threshold)	62 (12 %)	16 (4%)	46 (45%)

*Note*: SD, standard deviation.

Time until the first registered diagnosis of an SSD in specialized health care from the index hospitalization discharge date was predicted using Cox proportional hazards regression with R package *survival* (Therneau, 2023). Diagnosis of SSD was predicted with subthreshold symptoms and threshold symptoms (with ‘no psychotic symptoms’ used as the reference group). The discharge date from the index hospitalization was used as the entry date. Exit dates were the date of first registered psychosis diagnosis, date of death, or date of administrated censoring (July 1, 2023), whichever came first. Unadjusted as well as sex and age-adjusted estimates were produced. As a supplementary analysis, we ran the models stratified by sex (Supplementary Table S1). If participants had missing data on any of the variables of interest, they were dropped from the analyses (three individuals were dropped because of missing values). All tests were two-tailed, with statistical significance set at *p* < 0.05.

Sensitivity, specificity, accuracy, positive predictive value (PPV), negative predictive value (NPV), positive likelihood ratio (LR+), and negative likelihood ratio (LR-) were calculated for the ability of the threshold psychotic symptoms to correctly identify those who did and those who did not receive a diagnosis of SSD using R package *epiR* (Stevenson et al., 2024). The cumulative incidences were visualized using R packages *tidycmprsk* (Sjoberg & Fei, 2024) and *ggsurvfit* (Sjoberg et al., 2024).

As supplementary descriptive information, we examined the previous psychiatric diagnoses of participants who had received previous psychiatric treatment (Supplementary Table S2). We also examined the distribution of the first diagnosis of SSDs among individuals who did not have an SSD diagnosis at baseline and assessed the lifetime prevalence of different SSDs within this group. These results are presented in Supplementary Table S3. All analyses were performed using R Statistical Software (R Core Team, 2023).

## Results

The initial study sample consisted of 508 adolescent inpatients aged 13 to 17, of whom 59% were female. Descriptive characteristics of the sample at baseline are presented in Table 1. Of the included sample, a slightly higher proportion was female (60%) compared to the excluded sample (54%). The mean age of the included sample was 15.4 years and the mean duration of admission was 17 days. There were 13 people who died during the follow-up and did not receive a diagnosis of SSD, and thus were censored. The most common baseline diagnoses were affective disorders (56%), conduct disorders (49%), and substance use disorders (42%). Out of the included sample, 11% (*n* = 45) of individuals had threshold psychotic symptoms, 17% (*n* = 68) had subthreshold psychotic symptoms and 72% (*n* = 288) did not have any psychotic symptoms.

Over half of the included sample (*n* = 237, 59%) had previously received treatment in specialized psychiatric care and 112 (28%) had at least one prior psychiatric inpatient admission. Previous psychiatric diagnoses of the included sample are available in Supplementary Table S2.

**Figure 1.**
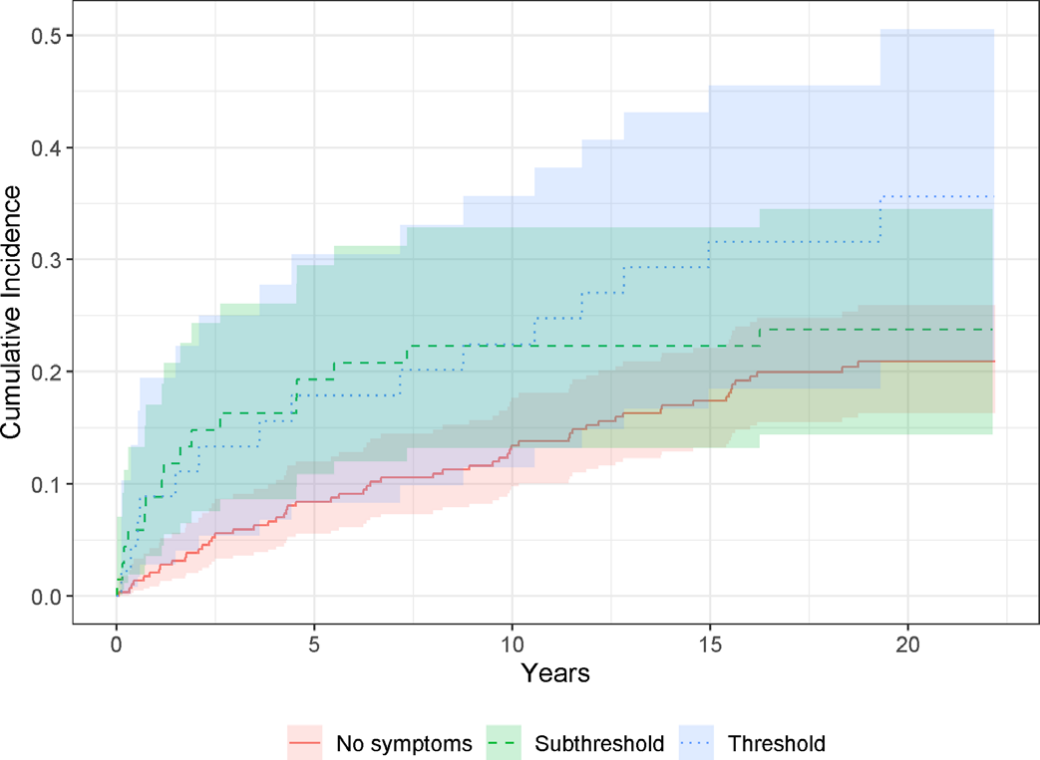
Cumulative incidence of diagnosis of psychosis during the follow-up stratified by the presence of psychotic symptoms. Time is displayed in years.

Table 2 presents the cumulative incidences of diagnoses of SSDs across symptom score categories among the included sample. Of those without subthreshold or threshold psychotic symptoms, 20% went on to be diagnosed with an SSD by the end of follow-up. Out of all individuals who received a diagnosis of an SSD by the study endpoint, 65% had not reported threshold or subthreshold psychotic symptoms at baseline assessment. The cumulative incidence of SSDs, stratified by the presence of psychotic symptoms, is illustrated in Figure 1.

**Table 2.** Distribution of baseline psychotic symptoms during adolescence stratified by the presence of an SSD diagnosis in specialized health care between participant’s discharge from index hospitalization and June 2023

	Diagnosed SSD (*n* = 91, 23%)			No diagnosed SSD (*n* = 313, 77%)		
	*n*	Psychotic symptoms at baseline (%)	Diagnosis of SSD (%)	*N*	Psychotic symptoms at baseline (%)	No diagnosis of SSD (%)
Psychotic symptoms						
1 (Not present)	58	65%	20%	230	74%	80%
2 (Subthreshold)	16	18%	24%	52	17%	76%
3 (Threshold)	15	17%	33%	30	10%	67%

*Note*: SSD, schizophrenia spectrum disorder.

Table 3 presents the results of the Cox regression model predicting the time until diagnosis of an SSD with threshold and subthreshold psychotic symptoms at baseline. Adolescents who reported subthreshold psychotic symptoms were not more likely to receive an SSD diagnosis by the end of follow-up (24%; adjusted HR = 1.42, 95% CI = 0.81–2.50). Adolescents who reported threshold psychotic symptoms were more likely to receive an SSD diagnosis by the end of follow-up (33%; adjusted HR = 2.00, 95% CI = 1.12–3.56).

**Table 3.** Results of the Cox regression predicting time to diagnosis of SSD with subthreshold and threshold psychotic symptoms

Model	HR (95% CI)	*p* value
Unadjusted model		
Subthreshold symptoms (ref. no symptoms)	1.26 (0.73–2.19)	0.411
Threshold symptoms (ref. no symptoms)	1.82 (1.03–3.20)	**0.040**
Age and sex adjusted model		
Subthreshold symptoms (ref. no symptoms)	1.42 (0.81–2.50)	0.223
Threshold symptoms (ref. no symptoms)	2.00 (1.12–3.56)	**0.019**
Female (ref. male)	0.58 (0.38–0.89)	**0.012**
Age	1.21 (1.03–1.43)	**0.024**

*Note:* CI, confidence interval; HR, hazard ratio; SSD, schizophrenia-spectrum disorder.

Bold values indicate a significance level of *p* < 0.05.

Threshold psychotic symptoms predicted future SSD with a sensitivity of 17% (95% CI: 10%–26%) and a specificity of 90% (95% CI: 87%–93%), with an accuracy of 74% (PPV: 33%, NPV: 79%, LR+: 1.75, LR−: 0.92). Sex-stratified analyses (see Supplementary Table S1) suggested the effect was driven by males, with threshold psychotic symptoms predicting subsequent SSD in male, but not female, patients.

## Discussion

We carried out a 17-year longitudinal study examining the association between psychotic symptoms in an inpatient adolescent psychiatry sample and subsequent schizophrenia-spectrum disorders. In total, 23% of inpatients admitted with non-psychotic mental disorders were diagnosed with an SSD by the study endpoint, highlighting that this is an important patient population in terms of psychosis prediction and prevention.

The risk of SSD did not differ significantly between patients with subthreshold psychotic symptoms and patients with no psychotic symptoms: 24% of the former group went on to be diagnosed with an SSD compared to 20% of the latter group. This highlights that inpatient psychiatry samples automatically have a high degree of pre-test risk enrichment for psychotic disorders, before any measures of attenuated psychotic symptoms are applied to them.

Based on visual inspection of the cumulative incidence curve (Figure 1), it appears that subthreshold and threshold psychotic symptoms might be associated with a more imminent risk of developing an SSD, based on the sharp incline in both curves from the point of study entry. This is in keeping with research on clinical high-risk (CHR) samples, where most of the risk for psychosis appears to be within the first 1–2 years following CHR diagnosis (Oliver et al., 2022). The slope of the curve for individuals who did not report (subthreshold or threshold) psychotic symptoms, however, is more gradual, suggesting that this group was further upstream of psychotic illness.

There was an increased risk of subsequent SSD diagnosis in patients who had reported full threshold psychotic symptoms during their initial inpatient admission: 33% of this group went on to receive an SSD diagnosis by the study endpoint. This suggests that threshold psychotic symptoms may have some utility in predicting later psychotic disorders in inpatient settings. It is notable, at the same time, that, of all SSDs diagnosed in this cohort, just 17% occurred in the group who had full threshold psychotic symptoms, meaning that 83% of future SSDs in this group would be missed if we were to focus only on individuals with full threshold psychotic symptoms.

In the sex-stratified models, the effect of threshold symptoms on future diagnosis of SSD was only significant for males. Although previous studies have had mixed findings regarding the effect of sex on the transition to psychosis in high-risk samples, there is some evidence that males might have a higher risk compared to females (Barajas et al., 2015). Our results support this, but more research is needed to draw further conclusions on the role of sex on transition in high-risk samples.

Our inpatient findings are in keeping with previous outpatient research by Lindgren et al. (2014) (Lindgren et al., 2014), who assessed psychotic symptoms among 161 young people attending outpatient adolescent psychiatry services in Finland using the Structured Interview for Psychosis Risk Syndromes (SIPS). They did not find evidence that clinical high-risk (CHR) criteria, based on SIPS assessments of psychotic symptoms, predicted psychotic disorders at 12-month follow-up in psychiatric outpatients, although the number of patients who developed a psychotic disorder in that cohort was low.

One other study investigated psychotic symptoms as a predictor of later psychotic disorders in child and adolescent psychiatric services in Italy (Mensi et al., 2021). In total, 212 adolescents were assessed for psychotic symptoms using the Comprehensive Assessment of at Risk Mental States (CAARMS) in child and adolescent neuropsychiatric services. The researchers found that young people with attenuated psychotic symptoms had an increased risk of psychotic disorder when followed for up to 5 years. Importantly, however, this study involved a mixed sample of both inpatients and outpatients and the psychotic symptom group was mainly (88%) comprised of psychiatric inpatients. Previous research has shown that young people with a history of inpatient psychiatric admission have a higher risk of subsequent psychotic disorder than those who have only attended outpatient services (Lång et al., 2022). This difference in pre-test risk enrichment associated with inpatient psychiatry services may explain the higher risk of psychotic disorder in the psychotic symptom group in that study.

While psychotic symptoms had limited utility in identifying risk for subsequent psychotic disorders in the current study, it may be the case that other clinical and demographic factors could help to identify those at greatest risk for psychosis in an inpatient setting. This might include information on, for example, family history (Rasic et al., 2014), non-psychotic psychopathology (van Os & Guloksuz, 2017), urbanicity (Empson et al., 2020), deprivation (O’Donoghue et al., 2016), adversity (Varese et al., 2012) and other clinical, demographic and social factors. Further research will be necessary to investigate this.

### Strengths and limitations

Key strengths of our study include (1) a high participation rate (83.7%), (2) the use of a combination of clinical and register data, allowing us to draw information from multiple sources, (3) a follow-up period of over 17 years, and (4) no attrition.

While the K-SADS-PL semi-structured interview is considered a gold standard for the assessment of psychopathology in children and adolescents, it is possible that additional psychotic-like symptoms might have been elicited if other clinical interviews were used, such as the SIPS or CAARMS. Notably, however, the proportion of inpatients with psychotic symptoms in this study (28%) was similar to the proportion meeting psychosis risk criteria in a previous cross-sectional adolescent inpatient psychiatry study that used the SIPS (26%) (Salazar de Pablo et al., 2020).

As participants were followed up in national healthcare registers, we only have information about diagnoses given in public specialist health care, which does not necessarily reflect the true incidence of SSDs in the population. However, as SSDs are almost always treated within specialist healthcare services in Finland and as there are no private psychiatric hospitals, these figures are likely to be a close reflection of the true population incidence.

As patients who had very brief inpatient admissions were less likely to have the opportunity to take part in the study, due to time constraints, it may have been the case that excluded patients disproportionately skewed towards individuals with milder illnesses (who had briefer admissions) compared to those who did take part in the study. We do not see, however, how this would have biased results on the relationship between psychotic symptoms and subsequent SSD. Finally, even though the high rate of participation is a strength of our study, we cannot rule out the possibility that the 16% of patients who did not take part may have differed in some way from the 84% who did.

## Conclusion

Schizophrenia-spectrum disorders are a common outcome for individuals with a history of adolescent psychiatry inpatient admission. Subthreshold psychotic symptoms did not help to differentiate those who would ultimately develop SSDs from those who would not. Threshold psychotic symptoms, on the other hand, were associated with an increased risk of SSDs by the study endpoint. Most SSDs, however, emerged in individuals who did not report either subthreshold or threshold psychotic symptoms at baseline, suggesting that interview assessments of psychotic symptoms may have somewhat limited utility for the detection of psychosis risk in inpatient settings. Our findings should inform the future use of psychosis risk measures in clinical services. Our findings also highlight, given the high risk for SSDs in this population, the need for future research in inpatient settings to investigate whether other factors might help to identify individuals at greatest risk of future psychosis, as well as research on how to reduce psychosis risk in this population.

## Supporting information

Kieseppä et al. supplementary material Kieseppä et al. supplementary material

## Supplementary material

The supplementary material for this article can be found at http://doi.org/10.1017/S003329172500073X.
