# Supplementary material for: Do psychotic symptoms predict future psychotic disorders in adolescent psychiatry inpatients? A 17-year cohort study
Source: Psychol Med. 2025 Apr 3;55:e108. doi: 10.1017/S003329172500073X (PMC12094655; doi:10.1017/S003329172500073X)
Supplement: Kieseppä et al. supplementary material [file S003329172500073Xsup001.zip › Table S1.docx]

| Table S1. Results of the Cox regression predicting time to diagnosis of SSD with subthreshold and threshold psychotic symptoms stratified by sex | | | | |
| --- | --- | --- | --- | --- |
|  | Male (n=158) | | Female (n=244) | |
| Variable | HR (95% CI) | p value | HR (95% CI) | p value |
| Unadjusted model |  |  |  |  |
| Subthreshold symptoms (ref. no symptoms) | 1.51 (0.59-3.87) | 0.393 | 1.37 (0.68-2.78) | 0.378 |
| Threshold symptoms (ref. no symptoms) | 2.66 (1.11-6.37) | **0.028** | 1.74 (0.81-3.71) | 0.153 |
| Age adjusted model |  |  |  |  |
| Subthreshold symptoms (ref. no symptoms) | 1.59 (0.62-4.09) | 0.335 | 1.34 (0.66-2.72) | 0.412 |
| Threshold symptoms (ref. no symptoms) | 2.55 (1.06-6.10) | **0.036** | 1.71 (0.80-3.65) | 0.166 |
| Age | 1.30 (1.02-1.66) | **0.032** | 1.13 (0.89-1.44) | 0.304 |
| *Note*. SSD = schizophrenia-spectrum disorder; HR = hazard ratio; CI = confidence interval. Bold values indicate significance level of p < 0.05 | | | | |
