# Supplementary material for: Do psychotic symptoms predict future psychotic disorders in adolescent psychiatry inpatients? A 17-year cohort study
Source: Psychol Med. 2025 Apr 3;55:e108. doi: 10.1017/S003329172500073X (PMC12094655; doi:10.1017/S003329172500073X)
Supplement: Kieseppä et al. supplementary material [file S003329172500073Xsup001.zip › Table S2.docx]

| Table S2. Distribution of psychiatric diagnoses in specialised health care prior to index admission among participants without a baseline or a previous diagnosis of psychosis who had previously received psychiatric treatment (n = 237) | | |
| --- | --- | --- |
| Diagnosis | n | % |
| Substance use disorders (F10-F19) | 17 | 7 % |
| Mood disorders (F30-F39) | 90 | 38 % |
| Anxiety disorders (F40-F49) | 36 | 15 % |
| Eating disorders and other behavioural syndromes (F50-F59) | 8 | 3 % |
| Disorders of adult personality and behaviour (F60-69) | 8 | 3 % |
| Pervasive and specific developmental disorders (F80-F89) | 9 | 4 % |
| Childhood behavioural and emotional disorders (F90-F98) | 102 | 43 % |
| *Note*. Only diagnoses given in specialised psychiatric health care from 1996 onwards are included. | | |
