# Supplementary material for: Do psychotic symptoms predict future psychotic disorders in adolescent psychiatry inpatients? A 17-year cohort study
Source: Psychol Med. 2025 Apr 3;55:e108. doi: 10.1017/S003329172500073X (PMC12094655; doi:10.1017/S003329172500073X)
Supplement: Kieseppä et al. supplementary material [file S003329172500073Xsup001.zip › Table S3.docx]

| Table S3. Distribution of psychosis diagnoses among those who did not have a baseline psychotic disorder but later received a psychosis diagnosis (n = 91) | | | | |
| --- | --- | --- | --- | --- |
|  | First diagnosis of a psychotic disorder* | | Lifetime diagnoses of psychotic disorders | |
| Diagnosis | n | % | n | % |
| Schizophrenia (F20) | 9 | 10% | 25 | 28% |
| Other chronic psychotic disorders (F21, F22, F25) | 9 | 10% | 24 | 26% |
| Brief psychotic disorder (F23) | 8 | 9% | 18 | 20% |
| Other/unspecified psychotic disorder (F28, F29) | 66 | 72% | 75 | 82% |

*One individual had two different psychosis diagnoses from different visits/admissions on the same day
