# Supplementary figures and images for: Do psychotic symptoms predict future psychotic disorders in adolescent psychiatry inpatients? A 17-year cohort study
Source: Psychol Med. 2025 Apr 3;55:e108. doi: 10.1017/S003329172500073X (PMC12094655; doi:10.1017/S003329172500073X)

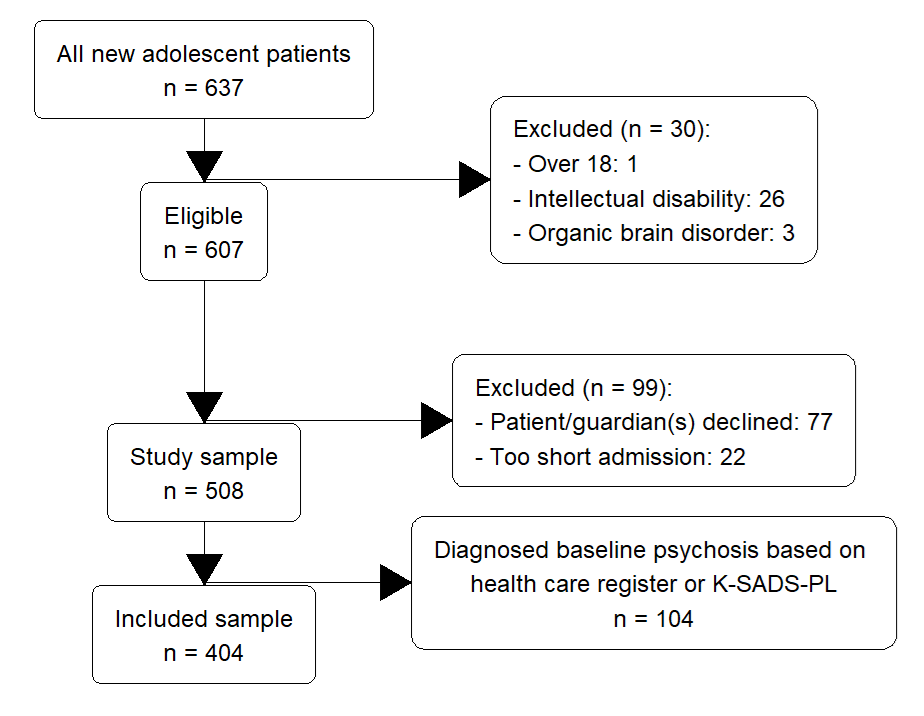


Figure S1. Flow chart of the participant selection process

Supplement: Kieseppä et al. supplementary material [file S003329172500073Xsup001.zip › Figure S1.docx]
